# Measuring PROMIS pain interference in German patients with chronic conditions: calibration, validation, and cross-cultural use of item parameters

**DOI:** 10.1007/s11136-023-03446-6

**Published:** 2023-06-02

**Authors:** Alexander Obbarius, Christoph Paul Klapproth, Gregor Liegl, Paula M. Christmann, Udo Schneider, Felix Fischer, Matthias Rose

**Affiliations:** 1 Department of Psychosomatic Medicine, Center for Internal Medicine and Dermatology, Charité – Universitätsmedizin Berlin, Berlin, Germany; 2 Dornsife Center for Self-Report Science, University of Southern California, Los Angeles, USA; 3 Department of Rheumatology and Clinical Immunology, Center for Internal Medicine and Dermatology, Charité – Universitätsmedizin Berlin, Berlin, Germany; 4 Quantitative Health Sciences, Outcomes Measurement Science, University of Massachusetts Medical School, Worcester, MA USA

**Keywords:** Pain interference, PROMIS, Item-response theory, Differential item functioning, Health-related quality of life, Rheumatology, Psychosomatic medicine

## Abstract

**Purpose:**

To calibrate the item parameters of the German PROMIS® Pain interference (PROMIS PI) items using an item-response theory (IRT) model and investigate psychometric properties of the item bank.

**Methods:**

Forty items of the PROMIS PI item bank were collected in a convenience sample of 660 patients, which were recruited during inpatient rheumatological treatment or outpatient psychosomatic medicine visits in Germany. Unidimensionality, monotonicity, and local independence were tested as required for IRT analyses. Unidimensionality was examined using confirmatory factor analyses (CFA) and exploratory factor analysis (EFA). Unidimensional and bifactor graded-response IRT models were fitted to the data. Bifactor indices were used to investigate whether multidimensionality would lead to biased scores. To evaluate convergent and discriminant validity, the item bank was correlated with legacy pain instruments. Potential differential item functioning (DIF) was examined for gender, age, and subsample. To investigate whether U.S. item parameters may be used to derive *T*-scores in German patients, *T*-scores based on previously published U.S. and newly estimated German item parameters were compared with each other after adjusting for sample specific differences.

**Results:**

All items were sufficiently unidimensional, locally independent, and monotonic. Whereas the fit of the unidimensional IRT model was not acceptable, a bifactor IRT model demonstrated acceptable fit. Explained common variance and Omega hierarchical suggested that using the unidimensional model would not lead to biased scores. One item demonstrated DIF between subsamples. High correlations with legacy pain instruments supported construct validity of the item bank. *T*-scores based on U.S. and German item parameters were similar suggesting that U.S. parameters could be used in German samples.

**Conclusion:**

The German PROMIS PI item bank proved to be a clinically valid and precise instrument for assessing pain interference in patients with chronic conditions.

**Supplementary Information:**

The online version contains supplementary material available at 10.1007/s11136-023-03446-6.

## Plain English summary

Pain can have significant impact on various areas of a persons’ health including on physical, emotional, and social aspects. Thus, to understand how pain impacts the life of individuals, it is important to assess this dimension in research and clinical settings. The PROMIS organization has developed an instrument (i.e. modern questionnaire) that is able to efficiently assess the impact of pain in individuals (“PROMIS Pain Interference item bank”). The items (questions) have been translated into several languages to allow for comparison of results across countries. The objective of this study was to investigate the psychometric properties of the German version of the PROMIS Pain Interference item bank. We used modern statistical methods (i.e. item-response theory), to investigate whether all items measure what they are supposed to measure. In addition, we investigated how precise (reliable) a measure of an individual is that answered all items or subsets of items. We found that the German version of the PROMIS Pain Interference item bank measures pain interference comparably to the original U.S. version and Dutch version suggesting that Pain Interference data can actually be compared across populations.

## Introduction

Reliable, valid, and precise assessment of pain states is key to effective treatment and follow-up of patients with chronic conditions [1]. Also, reliable and valid instruments are essential for pain assessments in clinical trials aiming at evaluating new treatments. Among the dimensions considered crucial for the assessment of pain is the impact of pain on individuals’ activities of daily living (‘pain interference’). Pain interference, sometimes also referred to as ‘pain impact’, includes consequences of pain such as reduced physical, social, or cognitive functioning as well as affected mental health or decreased quality of life [2]. Previous pain interference instruments such as the pain disability index (PDI) or the Brief Pain Inventory (BPI) have been widely used but exhibit certain limitations such as imprecise measurement of individual scores or the large number of items [3].

To overcome imprecise measurement and allow instrument-independent measurement, the Patient-Reported Outcomes Measurement Information System® (PROMIS®) has been developing tools for the assessment of a wide range of relevant health domains including pain intensity, pain interference, pain behavior, and pain quality [4–6]. Due to the use of item-response theory (IRT) models in the development of the PROMIS instruments, comparable measurements can be obtained using different subsets of items. This principle allowed the development of several abbreviated short-forms and computer-adaptive tests. Thus, only the most relevant items can be utilized in a test and eventually, patient burden is lower (lower number of items) while measurement precision is higher compared to conventional measures. This allows valid statements not only about health assessments of populations but also about individuals in clinical care [7].

The original English version of the PROMIS Pain Interference (PROMIS PI) item bank was developed and validated in a large combined sample of over 13.000 participants including a general population sample, cancer sample and chronic pain sample in the United States [8]. Follow-up studies confirmed and extended these findings in several populations [9–12]. The instrument has already been translated into several other languages including Spanish, Hebrew, Dutch, French, Portuguese, Korean, Nepali, Arabic, and German [13]. Whereas the first PROMIS PI item bank included 41 items (v 1.0), one item (“How often did pain make simple tasks hard to complete?”, PAININ39) was removed and a 40 item version (v 1.1) has been recommended for implementation. The first validation study of the German PROMIS PI in *n* = 262 patients undergoing rehabilitation could not confirm the unidimensional structure of the German PI items. Specifically, neither a unidimensional model, nor a bifactor model showed satisfactory model fit for further IRT analysis. Thus, based on exploratory factor analysis (EFA), the authors recommended a three-scale static measure (Pain Interference – German, PI-G) including a mental, physical, and functional subscale. Because of weak factor loadings, 13 items were removed so that the PI-G included a reduced set of 28 items [14]. These results contradict other validation studies of PROMIS PI translations, in which the unidimensional structure of the PI item bank was largely confirmed [15–17].

In the present study, we aim to investigate whether the German PROMIS pain interference items meet the assumptions for IRT analyses including unidimensionality, local independence and monotonicity. Because previous studies successfully fitted IRT models for 40 pain interference items [15–17], we aim at calibrating item parameters in a German sample of patients with chronic conditions. Furthermore, we examine the psychometric properties such as construct validity, differential item functioning, and measurement precision of the full item bank as well as the 4- and the 8-item short-forms. This study also investigates whether item parameters provided by PROMIS that were calibrated in U.S. samples can be used for estimating individual scores in a German sample. This is an important question, given the recommendation by the PROMIS health organization that the U.S. item parameters should be used globally.

## Materials and methods

### Setting, sample, and data collection

We analyzed data from a convenience sample of 660 patients. 214 patients were undergoing inpatient treatment at the Department of Rheumatology and Clinical Immunology at Charité and 446 patients were evaluated for inpatient treatment in the outpatient clinic at the Department for Psychosomatic Medicine at Charité. Rheumatology patients were recruited between September 2018 and August 2019 and Psychosomatic Medicine patients were recruited between August 2020 and May 2022. 446 Cases from the psychosomatic medicine clinic are a subsample of a larger assessment that aimed to evaluate a clinical routine assessment set. Cases were only used for data analyses in the present study if they had answered the question “Did you have any pain in the last 7 days?” with “yes”. Following informed consent, the 40 items of the German PROMIS PI adult item bank v1.1 were administered to the patients together with additional measures including a combination of PROMIS short-forms. Patients were excluded if they had already participated in the study during an earlier inpatient stay or if they were not able to understand the content of the questionnaires due to cognitive impairment or insufficient language skills.

### Measures

The original U.S. version of the PROMIS PI item bank v1.0 (41 items) was developed as part of the NIH funded PROMIS project and covers emotional, physical, and social impact of pain. [4]. The item bank was calibrated in a large U.S. sample including a general population sample, as well as clinical samples of cancer patients and patients with chronic pain [8]. Whereas the first PROMIS PI item bank included 41 items (v 1.0), one item (“How often did pain make simple tasks hard to complete?”, PAININ39) was removed and a 40-item version (v 1.1) has been recommended for implementation. The items have been translated into German by Farin et al. [14] according to the standard PROMIS methodology and were approved by the PROMIS Statistical Center [18].

We collected further measures to evaluate convergent and discriminant validity of the PROMIS Pain Interference item bank. Convergent validity was evaluated with three widely used pain interference/disability instruments: The Brief pain inventory (BPI, 7 items, range 0 to 10, higher scores indicate greater impairment) [19], Pain disability index (PDI, 7 items, range 0 to 10, higher scores indicate greater impairment) [20], and Owestry disability index (ODI, 10 items, range 0–5, greater scores indicate greater impairment) [21]. The Regional pain scale (RPS, 19 items, range 0 to 3, higher scores indicate greater dissemination and severity across the body) [22], PROMIS Pain Intensity 3a Scale v1.0 (3 items) as well as instruments for the assessment of other aspects of Health-related quality of life (HrQOL) including the EQ-5D-5L visual analogue scale on general health (1 item, range 0 to 100, greater scores indicate better health) [23], the PROMIS physical function short-form 4a v2.0, the PROMIS anxiety short-form 4a v1.0, the PROMIS depression short-form 4a v1.0, the PROMIS fatigue short-form 4a v1.0, and the PROMIS sleep disturbance short-form 4a v1.0 (www.healthmeasures.net) were used to evaluate discriminant validity of the PROMIS PI item bank. All PROMIS scores are reported on the T-Scores metric, where 50 represents the mean of the U.S. general population with a standard deviation of 10. Higher T-Scores indicate greater impairment (pain interference, anxiety, depression, fatigue sleep disturbance) or, in the case of physical function, greater functional ability.

### Statistical analyses

The analyses were carried out in accordance with similar studies and the PROMIS recommendations for item bank development [18, 24]. The software packages Mplus 8.4 [25], and R 4.2.1 [26] were used for analyses and visualization. R packages included mirt [27], mirtCAT [28], lavaan [29], lordif [30], mokken [31], MplusAutomation [32], psych [33] and ggplot2 [34].

#### Dimensionality of the item bank

A key assumption for estimating an IRT model is sufficient unidimensionality [7]. In accordance with PROMIS recommendations, the 40 PI items were first tested for unidimensionality using confirmatory (item-level) factor analysis (CFA). In the absence of strict unidimensionality, essential unidimensionality was examined with an array of exploratory factor analysis (EFA) models. [24]. A confirmatory approach is suggested as a first step because in the process of the item bank development, each potential pool of items (i.e. including the PI item pool) was carefully selected by experts to represent a dominant PRO construct through an exhaustive literature review and feedback from patients through focus groups and cognitive testing [8, 18, 24]. To account for the ordered categorical data, the weighted least square mean and variance adjusted (WLSMV) estimator was used for model estimation. To determine model fit, we used established criteria such as the Comparative Fit Index (CFI, cutoff > .95), the Tucker-Lewis Index (TLI, cutoff > .95), the Root Mean Square Error of Approximation (RMSEA, cutoff < .08), and the Standardized Root Mean Square Residual (SRMR, cutoff < .08) [18, 35]. Scaled indices were used to evaluate the fit. EFA including screeplot [36] and parallel analysis [37] was used to determine, whether the pool of items were sufficiently unidimensional. Recommended criteria suggest that sufficient unidimensionality is present, if 1) the first factor accounts for at least 20% of the variance, and 2) the ratio of eigenvalues between the first and subsequent factors exceeds 4 [24].

#### IRT model and item bank properties

We estimated a unidimensional and several multidimensional IRT models including bifactor IRT models. The factor structure of these confirmatory models was based on the EFA described above. Specifically, items were allocated to factors based on the highest factor loadings and based on a loading cut-off of ≥ 0.2 or ≤ − 0.2. To assess whether the bifactor models demonstrated sufficient unidimensionality that permit using a unidimensional IRT model instead, we used bifactor indices that have been suggested as viable for this specific purpose, i.e. Explained Common Variance (ECV) > 0.6, Omega hierarchical (OmegaH) > 0.8, and percentage of uncontaminated correlations (PUC) > 0.7 [38]. In compliance with PROMIS recommendations, Graded-Response Models (GRM) were applied for estimating IRT models [24, 39].

Further important assumptions for unidimensional IRT models are local independence and monotonicity [7]. Items are locally dependent if they show substantial correlations after correction for the common factor. Residual correlations of *r* > .25 were considered meaningful. The monotonicity assumption indicates that the probability of a correct response increases with increasing level on the latent trait. Monotonicity was evaluated using Mokken analysis [31]. Common rule of thumb criteria suggest Mokken H(i) to be ≥ .3 (weak) or ≥ .5 (strong) [40].

Model fit statistics were reported based on the M2* statistic [41]. The S−*Χ*^2^ fit statistic was calculated to investigate item fit to the model, comparing the expected and observed frequencies of the item category responses. Based on recommendations and earlier studies, a p(S−*X*^2^) value < .001 was chosen to indicate misfit to the IRT model [15, 16, 24]. Item parameters (slope and thresholds) were derived for the model. Discrimination (or, “slope”) refers to the ability of an item to differentiate among people with high pain interference and low pain interference. Or in other words, the larger the parameter, the more information about the localization on the latent trait the item can contribute. Threshold parameters represent the intersections of the probability functions of two item response curves. At this location on the latent trait, the probability of a person to respond to the higher or lower response category is equal (0.5 each). Thus, the item thresholds represent the spread of the item categories across the latent trait.

Factor scores (thetas) and corresponding standard errors for each person were estimated and converted into T-Scores by linear transformation (T-Score = [theta × 10] + 50). Measurement precision (standard error of measurement) and corresponding reliability across the T-Score continuum for the whole item bank as well as for the pre-defined 4-item and 8-item short-forms (PROMIS PI short-form 4a/8a v1.1, www.healthmeasures.net) were calculated.

#### Qualitative comparisons between German and U.S. models

To investigate whether item parameters estimated in our sample were comparable to original U.S. parameters, we evaluated the similarity of German and U.S. models, item parameters, and resulting *T*-Scores. To account for sample specific differences of the IRT models, the Stocking-Lord test characteristic curve equating procedure [42] was used to determine linear transformation constants that allow to align the newly estimated German model with the previously published U.S. model (www.assessmentcenter.net). Item characteristic curves (ICC) and test characteristic curves (TCC) of both models were compared to each other. Differences between ICCs and TCCs were plotted and inspected. Outlier items (i.e. items that showed a pronounced difference in the ICC curves between both models) were identified. Pearson correlations were used to evaluate the similarity of T-Scores based on the original U.S. model and newly estimated German model. Bland–Altman plots were used to illustrate the agreement between T-Scores based on item parameters that were calibrated in the German and U.S. samples (each for the full item bank and 4-, and 8-item short-forms) [43]. In addition to bias (i.e. deviation of the average difference from zero), and lower and upper limits of agreement (i.e. within which 95% of the differences fall), mean absolute error was used to describe the average disagreement (i.e. regardless of the direction) between corresponding T-Scores based on the U.S. and German models.

#### Differential item functioning

Items in an item bank should ideally perform equally among different groups such as age groups or gender [24]. To avoid bias, the probabilities of deriving certain item responses need to be independent of subgroup membership [44]. We examined potential differential item functioning (DIF) of age, gender, and subsample (Rheumatology versus Psychosomatic medicine sample). DIF testing was based on a unidimensional model only. We used an iterative hybrid approach of ordinal logistic regression (OLR) and IRT as implemented in the lordif R-package [30]. This procedure was used to maintain high comparability with other studies that investigated DIF in PROMIS PI items [8, 15, 17, 45–47]. Specifically, for each item, the expected response based on latent ability and group membership is modeled. Next, regression models implying no DIF, uniform DIF, and non-uniform DIF are compared between groups based on a pseudo *R*^2^ measure [48]. If the *R*^2^ difference between models exceeds 0.03, items are flagged for uniform and/or non-uniform DIF [49]. This procedure is repeated until a stable set of items exhibiting DIF is identified. To identify age DIF, elderly (≥ 65 years) were compared with younger patients, because evidence suggests that elderly report pain differently than younger people [50]. For items that demonstrate DIF, clinical relevance was evaluated by comparing theta estimates based on non-group-specific item parameters with theta estimated based on the DIF-free and group-specific item parameters, obtained with lordif, using Pearson correlations and Bland–Altman plots [43].

#### Convergent and discriminant validity

PI T-scores based on the full item bank were correlated with above mentioned instruments. To account for non-normal distribution of the pain data, Spearman rank correlations were used [51]. We expected a high positive correlation of rho ≥ 0.6 between the PI T-Scores other PI instruments including BPI, PDI, and ODI. We expected a lower correlation with other theoretically different domains such as pain intensity, pain location, depression, anxiety, or physical function. Due to the conceptual overlap of the pain constructs [52] and due to the fact that there is a stable association between construct that reflect aspects of self-reported health [53], we expected medium correlations of 0.3 ≤ rho < 0.6 rather than lower correlations.

## Results

### Sample

Participant characteristics are provided in Table 1. On average, patients in the rheumatology sample were about 10 years older than in the psychosomatic medicine sample. In both samples, two-third were female, and more than half of the patients lived with a partner. About one-third in both samples had a master, bachelor, or doctoral degree. Whereas in the rheumatology patients, about 25% was working part- or fulltime, this was the case in about 60% of the psychosomatic medicine patients. More than half of the rheumatology patients had a connective tissue disease. The most frequent diseases in the psychosomatic medicine patients were depression (13.9%) and anxiety disorder (10.1%). In both samples, patients reported a medium pain level, reduced physical functioning, and elevated levels of anxiety, depression, fatigue, and sleep disturbance, compared with the general population.

**Table 1:** Sample characteristics

	Sample 1 (rheumatology) *n* = 214	Sample 2 (psychosomatic medicine) *n* = 446
Age in years, mean ± SD (range)	55.7 ± 16.9 (19–89)	44.1 ± 13.9 (18–82)
Gender, *n* (%)		
Female	137 (64.0)	282 (63.8)
Male	66 (30.8)	157 (35.5)
Non-binary		3 (0.7)
Living status, *n* (%)		
With partner	135 (63.1)	234 (52.9)
Single	63 (29.4)	170 (38.5)
Other	7 (3.3)	38 (8.6)
Educational level (ISCED 1997^a^), *n* (%)		
Doctoral or equivalent	5 (2.3)	
Bachelor/Master or equivalent	74 (34.6)	167 (38.3)
Degree of post-secondary/tertiary education	83 (38.7)	
Degree of secondary education	36 (16.8)	167 (38.1)
Degree of primary education	2 (0.9)	
Without	3 (1.4)	6 (1.4)
Work status, *n* (%)		
Full-time	29 (13.6)	172 (39.2)
Part-time	21 (9.8)	87 (19.8)
Seeking employment	3 (1.4)	23 (5.2)
Not employed (student, retired, freelancer)	79 (36.9)	105 (23.9)
Medical conditions^b^, *n* (%)		
Chronic pain (≥ 6 months)	156 (82.1)	40 (9.0)
Rheumatoid arthritis	55 (25.7)	
Connective tissue disease	123 (57.5)	
Vasculitis	36 (16.8)	
Osteoarthritis, spondylopathy	23 (10.7)	
Fibromyalgia, somatoform pain disorder	19 (8.9)	
Gastro-intestinal diseases		44 (9.9)
Depression		62 (13.9)
Anxiety disorder		45 (10.1)
Somatoform disorder		41 (9.2)
Instrument scores, M (SD)		
PROMIS pain intensity^c^	4.55 (2.45)	5.02 (2.10)
Brief pain inventory (BPI)^c^	3.28 (2.44)	
Owestry disability Index (ODI)^c^	3.12 (2.10)	
Pain disability Index (PDI)^c^	4.13 (2.71)	
Regional pain scale (RPS)^c^	2.48 (1.91)	
EQ-5D-5L General health^d^	5.6 (2.1)	
PROMIS Physical function^e,f^	36.5 (9.6)	43.3 (8.0)
PROMIS anxiety^e,g^	54.5 (9.2)	59.3 (10.0)
PROMIS depression^e,g^	56.0 (8.9)	60.0 (9.2)
PROMIS fatigue^e,g^	56.2 (9.6)	59.8 (9.5)
PROMIS sleep^e,g^	53.9 (9.1)	55.5 (8.4)

^a^International Standard Classification of Education;

^b^diagnoses are not mutually exclusive;

^c^range 0–10, 0 = no impairment, 10 = full impairment;

^d^0 = worst health, 10 = best health;

^e^PROMIS *T*-Score, general population mean = 50, standard deviation = 10

^f^Higher values = better physical functioning

^g^Higher values = higher severity

*n* Number; *SD* standard deviation

### IRT assumptions and model estimation

CFA of a one-factor model across 40 PI items did not result in acceptable fit (CFI = 0.91; TLI = 0.91; RMSEA = 0.128; SRMR = 0.08). The screeplot suggested a one-factor solution, whereas the parallel analysis suggested up to 5 factors. The eigenvalue of the first factor was 26.10, the eigenvalues of factors 2 to 5 were 2.11, 1.17, 0.74, and 0.54, respectively. The first factor accounted for 65.3% of the variance, the ratio of the eigenvalues of the first two factors was 12.3, which means that both values well exceeded the recommended criteria suggesting that there was sufficient unidimensionality for subsequent IRT analyses.

No item-pair showed local dependency, the highest residual correlation was *r* = 0.25. Mokken H(i) of the full PI item bank was 0.638, H(i) coefficients of the individual PI items were between 0.521 and 0.704 indicating strong scalability, i.e. sufficient monotonicity. We concluded that the 40 PI items met the IRT assumptions.

The unidimensional IRT model did not indicate sufficient model fit. Whereas multidimensional IRT models with up to 5 factors did also not achieve recommended model fit cut-offs, bifactor models well exceeded the cut-offs (Table 2). The 4-factor bifactor model (one general factor and three specific factors) demonstrated the best fit. Bifactor indices suggested that a unidimensional model could be used instead of a bifactor model (Table 2). Only three items had an item-level ECV slightly below 0.6: PAININ55 (0.560), PAININ50 (0.583), PAININ11r1(0.598). Therefore, a unidimensional IRT model was used for calibration.

**Table 2:** Model fit statistics for graded-response item-response theory models in 40 pain interference items

Model	df	RMSEA (95% CI)	SRMR	TLI	CFI	ECV	IECV range	PUC	OmegaH
Uni-dimensional	620	0.112 (0.109–0.115)	0.07	0.89	0.90				
2-Factor	621	0.106 (0.103–0.109)	0.42	0.90	0.91				
4-Factor	614	0.132 (0.129–0.134)	0.49	0.85	0.86				
5-Factor	610	0.138 (0.135–0.140)	0.51	0.83	0.84				
2-Factor bifactor	599	0.079 (0.076–0.082)	0.06	0.94	0.95	0.92	0.69–1.00	0.73	0.99
3-Factor bifactor	596	0.080 (0.077–0.083)	0.06	0.94	0.95	0.88	0.51–1.00	0.82	0.98
4-Factor bifactor	589	0.074 (0.071–0.077)	0.05	0.95	0.96	0.85	0.56–1.00	0.79	0.97
5-Factor bifactor	587	0.078 (0.075–0.081)	0.06	0.95	0.95	0.83	0.54–1.00	0.81	0.98

*CI* Confidence Interval; *CFI* Comparative Fit Index; *df* degrees of freedom; *ECV* Explained Common Variance; *IECV* Item-level ECV; *OmegaH* Omega hierarchical; *M* Mean; *min* minimum; *RMSEA* Root Mean Square Error of Approximation; *SD* standard deviation; *SRMR* Standardized Root Mean Square Residual; *TLI* Tucker-Lewis Index

A graded response model was fitted to the data. Item characteristics including fit statistics as well as IRT parameters are provided in Table 3. There was no item with a *p*(S-X2) below 0.001, indicating satisfactory fit of all items in the IRT model. The item slope parameters (‘a’) ranged between 1.66 and 3.93, the item threshold parameters (‘b1’ to ‘b4’) ranged between -2.10 and 2.65. The item with the highest discrimination (steepest slope) was PAININ10 (“How much did pain interfere with your enjoyment of recreational activities?”).

**Table 3:** Item content and properties of the German PROMIS Pain interference item bank

Item^a^	Content	M	SD	Skewness	Item parameters					Item fit: p (S-X2)
					a	b1	b2	b3	b4	
PAININ1^b^	How difficult was it for you to take in new information because of pain?	2.33	1.19	0.55	1,82	− 0,63	0,32	1,20	2,23	0,546
PAININ3	How much did pain interfere with your enjoyment of life?	3.43	1.16	− 0.35	2,84	− 1,98	− 0,70	− 0,09	0,96	0,788
PAININ5	How much did pain interfere with your ability to participate in leisure activities?	3.29	1.23	− 0.31	3,70	− 1,38	− 0,57	0,09	0,96	0,463
PAININ6	How much did pain interfere with your close personal relationships?	2.78	1.28	0.16	3,17	− 0,90	− 0,09	0,49	1,34	0,612
PAININ8	How much did pain interfere with your ability to concentrate?	2.92	1.27	0.10	2,09	− 1,30	− 0,27	0,51	1,38	0,974
PAININ9^b.c^	How much did pain interfere with your day to day activities?	3.34	1.18	− 0.22	2,86	− 1,88	− 0,64	0,07	1,01	0,463
PAININ10	How much did pain interfere with your enjoyment of recreational activities?	3.26	1.26	− 0.29	3,93	− 1,30	− 0,48	0,06	0,93	0,702
PAININ11r1	How often did you feel emotionally tense because of your pain?	3.19	1.14	− 0.40	2,26	− 1,55	− 0,74	0,16	1,55	0,348
PAININ12^b^	How much did pain interfere with the things you usually do for fun?	3.43	1.20	− 0.39	3,11	− 1,72	− 0,68	− 0,06	0,90	0,781
PAININ13^b^	How much did pain interfere with your family life?	2.94	1.29	− 0.01	3,09	− 1,02	− 0,33	0,37	1,22	0,344
PAININ14	How much did pain interfere with doing your tasks away from home (e.g.. getting groceries. running errands)?	3.03	1.29	− 0.03	3,23	− 1,14	− 0,35	0,34	1,08	0,294
PAININ16	How often did pain make you feel depressed?	3.24	1.08	− 0.56	2,78	− 1,53	− 0,82	0,09	1,57	0,604
PAININ17	How much did pain interfere with your relationships with other people?	2.70	1.27	0.23	3,83	− 0,80	− 0,02	0,56	1,34	0,405
PAININ18	How much did pain interfere with your ability to work (include work at home)?	3.32	1.30	− 0.28	3,39	− 1,38	− 0,51	0,05	0,81	0,257
PAININ19	How much did pain make it difficult to fall asleep?	2.76	1.31	0.26	1,69	− 1,16	− 0,08	0,71	1,55	0,370
PAININ20	How much did pain feel like a burden to you?	3.76	1.16	− 0.73	2,73	− 2,10	− 1,04	− 0,41	0,57	0,016
PAININ22^b.c^	How much did pain interfere with work around the home?	3.01	1.23	− 0.03	3,04	− 1,25	− 0,36	0,34	1,25	0,668
PAININ24	How often was pain distressing to you?	3.51	1.04	− 0.69	2,89	− 1,88	− 1,12	− 0,17	1,20	0,793
PAININ26	How often did pain keep you from socializing with others?	2.71	1.21	0.00	3,38	− 0,80	− 0,17	0,55	1,74	0,804
PAININ29	How often was your pain so severe you could think of nothing else?	2.62	1.22	0.08	2,63	− 0,75	− 0,11	0,66	1,95	0,187
PAININ31^b.c^	How much did pain interfere with your ability to participate in social activities?	3.11	1.29	− 0.11	3,52	− 1,15	− 0,41	0,28	0,98	0,508
PAININ32	How often did pain make you feel discouraged?	2.84	1.20	− 0.08	2,28	− 1,09	− 0,33	0,51	1,82	0,080
PAININ34^b.c^	How much did pain interfere with your household chores?	2.95	1.24	0.03	2,88	− 1,21	− 0,29	0,39	1,30	0,923
PAININ35	How much did pain interfere with your ability to make trips from home that kept you gone for more than 2 h?	2.99	1.44	− 0.02	3,40	− 0,80	− 0,20	0,25	0,89	0,008
PAININ36^b^	How much did pain interfere with your enjoyment of social activities?	3.18	1.29	− 0.18	3,58	− 1,24	− 0,41	0,16	0,95	0,884
PAININ37	How often did pain make you feel anxious?	3.01	1.15	− 0.25	1,82	− 1,52	− 0,58	0,39	1,96	0,379
PAININ38	How often did you avoid social activities because it might make you hurt more?	2.62	1.29	0.15	2,83	− 0,63	− 0,05	0,60	1,67	0,345
PAININ40	How often did pain prevent you from walking more than 1 mile?	2.61	1.44	0.31	2,30	− 0,49	0,04	0,62	1,34	0,084
PAININ42	How often did pain prevent you from standing for more than one hour?	2.92	1.50	0.03	1,94	− 0,78	− 0,21	0,35	1,10	0,226
PAININ46	How often did pain make it difficult for you to plan social activities?	2.78	1.29	− 0.01	3,64	− 0,74	− 0,20	0,47	1,43	0,407
PAININ47	How often did pain prevent you from standing for more than 30 min?	2.76	1.47	0.15	2,00	− 0,63	− 0,10	0,45	1,28	0,809
PAININ48	How much did pain interfere with your ability to do household chores?	2.89	1.31	0.07	3,14	− 0,98	− 0,20	0,40	1,20	0,590
PAININ49	How much did pain interfere with your ability to remember things?	2.07	1.19	0.84	1,81	− 0,18	0,59	1,43	2,35	0,859
PAININ50	How often did pain prevent you from sitting for more than 30 min?	2.31	1.25	0.52	1,89	− 0,45	0,31	1,06	2,22	0,959
PAININ51	How often did pain prevent you from sitting for more than 10 min?	1.83	1.05	1.12	2,20	0,10	0,90	1,60	2,65	0,300
PAININ52	How often was it hard to plan social activities because you didn't know if you would be in pain?	2.62	1.34	0.19	2,77	− 0,57	− 0,05	0,60	1,55	0,092
PAININ53	How often did pain restrict your social life to your home?	2.72	1.25	0.05	3,52	− 0,77	− 0,12	0,52	1,59	0,151
PAININ54	How often did pain keep you from getting into a standing position?	2.21	1.44	0.81	1,66	− 0,03	0,52	1,02	1,65	0,855
PAININ55	How often did pain prevent you from sitting for more than one hour?	2.27	1.30	0.60	2,06	− 0,27	0,38	0,93	2,03	0,627
PAININ56	How irritable did you feel because of pain?	2.89	1.30	0.06	1,77	− 1,25	− 0,27	0,51	1,53	0,296

^a^Item names are following the PROMIS standards (www.healthmeasures.net)

^b^PROMIS PI 8-item short-form

^c^PROMIS PI 4-item short-form

*M* mean; *SD* standard deviation

### Qualitative comparisons between German and U.S. models

The coefficients for linear transformation of newly estimated item parameters were 0.696 (constant A) and 11.918 (constant B). When the ICCs of the U.S. model and newly estimated model were compared, the majority of the items were similar to each other (Figure S1 and S2, online supplemental material). The difference in expected item scores between the models, exceeded one score point (i.e. on a 5-point scale) for one item, PAININ40 (“How often did pain prevent you from walking more than 1 mile?”) whereas the differences for all other items was 0.6 points or less. However, differences between expected test scores of the full item bank (with and without PAININ40) and 4-item, and 8-item short-forms, were only small (Figure S2, online supplemental material) suggesting that differences for single items compensate each other and may be, at least in part, due to sampling error.

**Fig. 1:**
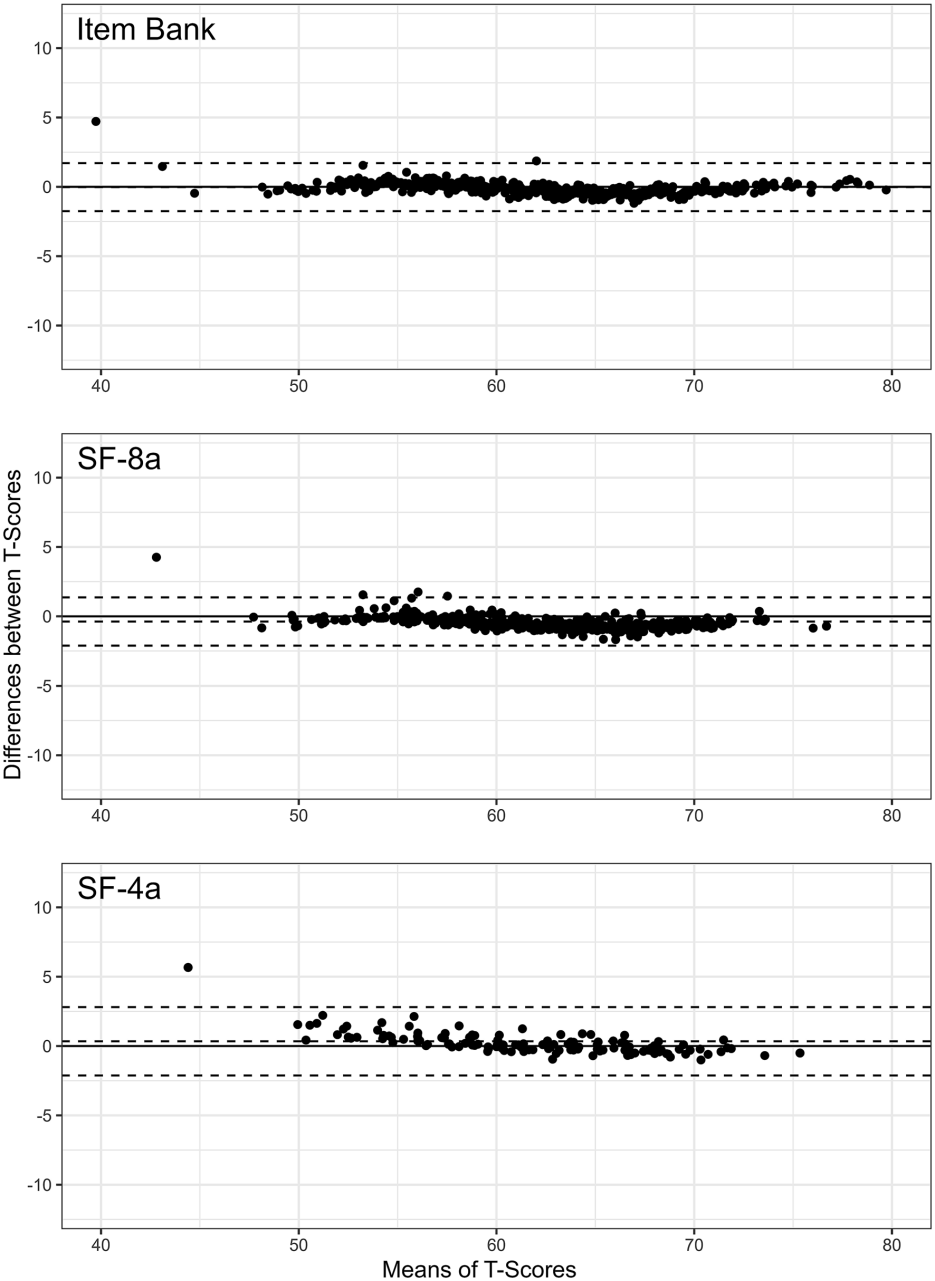
Agreement between German and U.S. IRT models. The Bland–Altman plots show the agreement between *T*-Scores based on item parameters which were calibrated in German patients with a range of chronic conditions, and *T*-Scores based on item parameters that were calibrated in a U.S. general population sample (www.assessmentcenter.net). The plots illustrate agreement of *T*-scores based on the 40-Item German PROMIS PI Item Bank v1.1, the 8-item short-form (SF-8a), and the 4-item short-form (SF-4a). The broken lines show mean scoring differences across the pain interference continuum as well as empirical 95% limits of agreement. The differences between the inner broken lines and solid lines indicate the small average biases between both theta calculation methods of − 0.024, − 0.378, and 0.342 for the full item bank, the 8-item short-form, and the 4-item short-form, respectively

Correlation analyses of the *T*-Scores obtained with the item parameters based on the German sample and *T*-Scores obtained with the item parameters based on the U.S. sample demonstrated high accordance for the full item bank (*r* = .995), as well as 8-item (*r* = .995) and 4-item (*r* = .993) short-forms. The agreement between *T*-Scores is illustrated in Fig. 1. The bias [lower limit of agreement, upper limit of agreement] for the full item bank, SF-8a, and SF-4a was − 0.02 [− 1.75, 1.71], − 0.38 [− 2.12, 1.36], and 0.34 [− 2.13, 2.81] T-Score points. The mean absolute error between corresponding T-Scores was 0.46 (item bank), 0.67 (SF-8a), and 0.63 (SF-4a). These findings confirm the high consistency of T-Scores based on the German and U.S. item parameters.

### Differential item functioning

None of the items showed DIF for gender or age, whereas item PI40 (“How often did pain prevent you from walking more than 1 mile?”) demonstrated DIF for subsample. PI40 resulted in higher *T*-Score values in the psychosomatic medicine sample compared to the rheumatology sample. However, the differences between corrected T-Scores and uncorrected T-Scores were very low, suggesting that sample specific item parameters for PI40 are not necessary. On average, T-Score differences were 0.038 (standard deviation = 0.027), the highest difference for an individual was 0.315 *T*-Score points.

### Item bank properties and convergent/discriminant validity

**Fig. 2:**
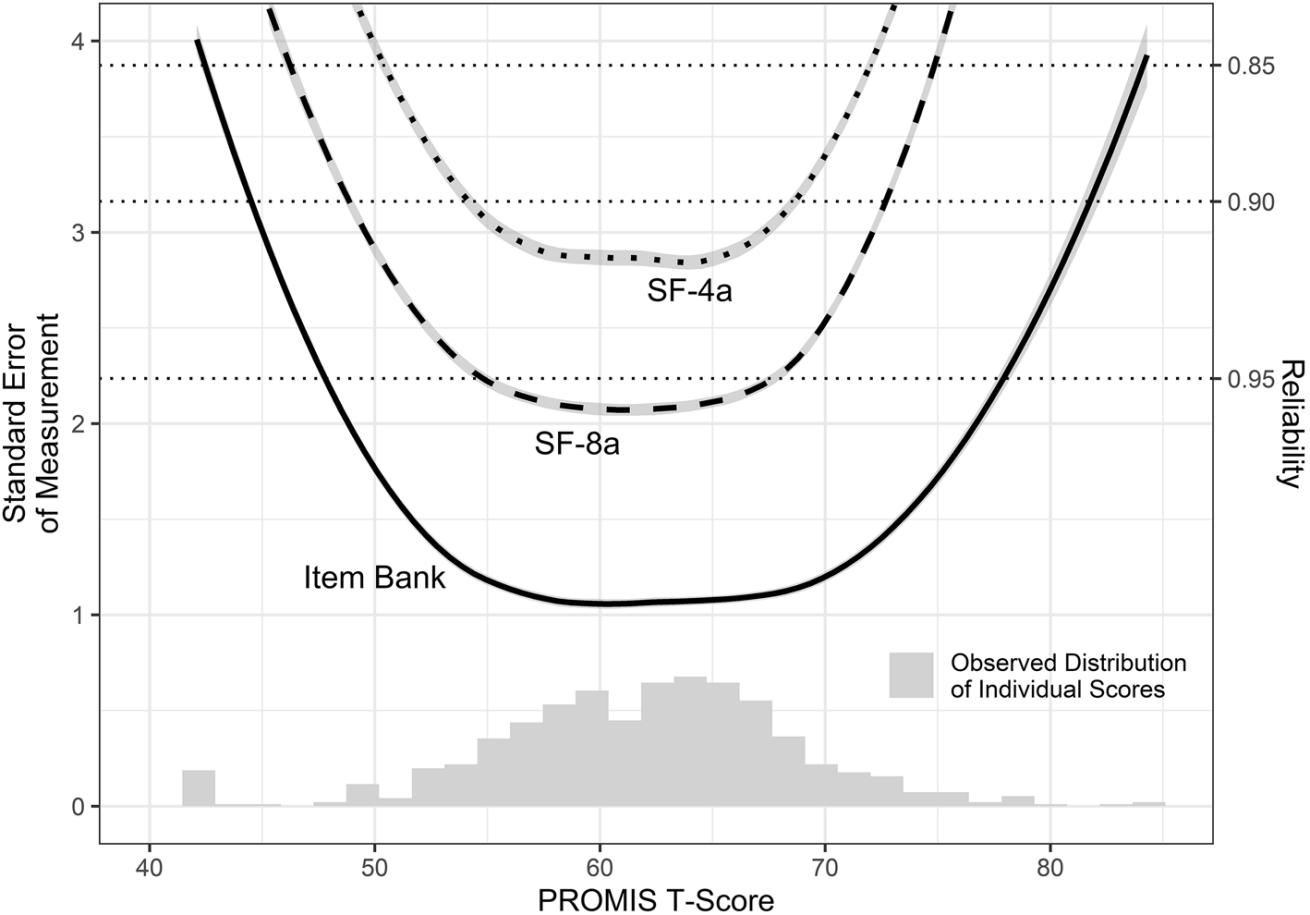
Precision of the PROMIS Pain Interference Item Bank and Short-Forms. Standard error of measurement and corresponding reliability across the latent Pain interference continuum of the 40-Item German PROMIS Pain Interference Item Bank v1.1 and derived 4-Item, and 8-Item Short-Forms (SF-8a, SF-4a) obtained in a sample of *n* = 660 rheumatology and psychosomatic medicine patients. A *T*-score of 50 represents the average of the U.S. general population, the standard deviation is 10. A lower *T*-score score corresponds to less “ability” on the latent trait (less interference due to pain), whereas a higher *T*-score corresponds to more “ability” on the latent trait (more interference due to pain)

The full item bank demonstrated high precision (SEM ≤ 3.2, corresponding to classical reliability of 0.9) on the T-Score continuum between 45 and 83 (Fig. 2). As expected, the range in which the short-forms measure with high precision was narrower. However, the short-forms demonstrated high precision on the T-Score metric between 55 and 70, where most scores are located.

The direction and size of correlations with other instruments supported the construct validity of the item bank (Table 4). Correlations with other instruments assessing aspects of pain interference such as BPI, ODI, and PDI were above 0.7 (convergent validity) and correlations with other measures assessing different aspects of pain (i.e. intensity, location) and health (depression, anxiety, physical functioning, fatigue, sleep disturbance) were between 0.4 and 0.6 (discriminant validity).

**Table 4:** Spearman’s rank correlations between the PROMIS pain interference item bank and other self-report measures

		PROMIS pain interference item bank^a^
Pain measures	Sample size (*n*)	rho
PROMIS pain interference SF-8a	658	.949
PROMIS pain interference SF-4a	654	.892
PROMIS pain intensity	214	.582
Brief pain inventory (BPI)	209	.716
Owestry disability index (ODI)	208	.736
Pain disability index (PDI)	208	.811
Regional pain scale (RPS)	203	.574
Other measures		
EQ-5D-5L VAS general health	209	− .448
PROMIS physical function SF-4a	647	− .544
PROMIS anxiety SF-4a	637	.438
PROMIS depression SF-4a	647	.496
PROMIS fatigue SF-4a	646	.521
PROMIS sleep SF-4a	629	.415

^a^Correlation between individual pain interference T-Scores obtained with the full item bank and other measures

*rho* Spearman correlation coefficient; *SF* Short-Form; *VAS* visual analogue scale

## Discussion

We investigated the psychometric properties of the German PROMIS PI item bank in 660 patients with chronic conditions. In contrast to a previous validation study of the German PROMIS PI items [14], the items demonstrated sufficient unidimensionality for IRT analyses and we successfully calibrated item parameters for all 40 German PROMIS PI items. The item bank as well as the 4-item and 8-item short-forms showed excellent measurement precision on a broad range of the latent pain interference continuum. This does not only allow for reliable group-based statements, for example in clinical trials, but also for reliable statements about individuals in clinical settings. In addition, we found that the item parameters calibrated in our German sample result in highly similar T-scores compared to T-scores that were obtained using the item parameters provided by PROMIS that were calibrated in U.S. samples. These results suggest that U.S. item parameters may be used in German populations, at least if they are consisting of chronically ill patients. This was an important finding, given the recommendation of the PROMIS Health Organization that the item parameters based on U.S. populations should be used globally (www.healthmeasures.net).

Other efforts on validating the PROMIS Pain interference item bank in other languages were similarly successful [8, 15–17]. For both the original U.S. version and the Dutch-Flemish version of the item bank, the authors found a sufficiently unidimensional structure and were able to calibrate item parameters for the 40 PROMIS PI items. Like in our study, however, the unidimensional CFA did not result in sufficient model fit. Three studies successfully used EFA to determine whether the PROMIS PI items were sufficiently unidimensional [8, 17, 46]. In those three studies, similar to the present study the first factor accounted for the vast majority (86, 66, and 79%) of the variance and the ratio of eigenvalues of the first and second factor well exceeded the recommended cut-off of 4 (35.3, 13.0, and 29.5). Another study that aimed to validate the PROMIS PI item bank in Dutch patients with musculoskeletal conditions also found suboptimal fit of a unidimensional model and used bifactor analysis instead. Similar to the present study, bifactor indices indicated that a unidimensional model represents the data sufficiently well [16]. Thus, although none of the studies that evaluated the PROMIS PI item bank – including the present study—did find that a unidimensional CFA demonstrated good fit, follow-up investigation using EFA and confirmatory bifactor analyses pointed at sufficient unidimensioniality.

The findings on the comparability of our IRT model with the original PROMIS model adds to the evidence on cross-cultural validity of PROMIS pain scales [15, 46, 54]. This allows, for example, direct comparison of PROMIS scores across countries in clinical trials or even clinical settinsg without controlling for country-specific differences. In contrast to some previous studies we did not aim at calculating DIF between populations because our sample was not well comparable with the PROMIS pain validation sample [8]. If DIF had been found, we would not have been able to differentiate whether the bias had been caused by culture- or sample-specific differences. Our findings on culture-specific differences can be attributed to sampling error – at least to a certain extend – because differences between ICCs show approximately normal distribution, except for one outlier, PAININ40 (“How often did pain prevent you from walking more than 1 mile?”). The reason may be that there is actual cross-cultural DIF because of the translation of this item into German because “1 mile” was translated as “1 km”, which is only about two-thirds of the distance.

To allow comparison between established instruments such as those mentioned above as well as other clinically used instruments such as the pain interference items of the German Pain Questionnaire [55] and PROMIS PI, future studies should aim at linking these items or instruments to the PROMIS metric. Several studies have been published that allow cross-linking between the English versions of PROMIS PI and other pain measures including BPI, SF-36 Bodily Pain Subscale, ODI, the pain interference item of the Patient-Reported Outcomes version of the Common Terminology Criteria for Adverse Events (PRO-CTCAE®), [56–59] but studies in other languages (including German) are pending. Given the finding that item parameters based on a German sample lead to highly similar scores to when item parameters calibrated in U.S. samples are used, it would be highly interesting to see if linking German versions of classical pain interference instruments (such as the BPI, PDI, or ODI) to the PROMIS metric would result in similar cross-links (i.e. item parameters and crosswalk scores) compared to the linking studies in U.S. populations.

In addition, data from the general population in German-speaking countries would allow to establish population-based T-Scores and to evaluate measurement invariance between sample subgroups and languages. A recent study found that the items from the PROMIS PI 4-item short-form are relatively measurement-non-invariant across general population samples from France, United Kingdom, and Germany although the authors note that there has to be some measurement bias taken into account when small effects between countries are investigated [54]. Thus, a general population sample would allow for evaluation of measurement invariance and identification of T-score differences between populations of the full German PROMIS PI item bank.

Strengths of this study include the confirmation of the unidimensional structure that is a fundamental requirement for item banking, the relevant clinical sample, and the evaluation of systematic language-specific differences of the PROMIS PI construct. A few limitations have to be mentioned: The sample size is smaller compared to the English and Dutch evaluation studies [8, 15] resulting in limited generalizability and statistical power. However, we exceeded the minimum sample size for IRT-based modeling of at least 500 patients recommended by general guidelines [60]. In addition, the sample was a convenience sample from a clinical population and results may be specific for this group of patients. Thus, evaluation in other clinical and non-clinical samples including the general population is necessary. Also, we calibrated the item parameters of a unidimensional IRT model, although fit statistics suggested that a 4-factor bifactor model represented the data best. The agreement between factor scores based on the bifactor IRT model and factor scores based on the unidimensional IRT model was very high (*r* = 0.999), however, differences in individual scores ranged between -1.57 and 1.72 on the T-Score metric. These differences are small given the standard deviation of 10 and will probably in most cases not be clinically relevant.

In conclusion, the German PROMIS PI item bank v.1.1 showed excellent measurement precision on a broad range of the latent construct. Thus, based on this item bank, computer-adaptive testing or short-forms could be used for precise assessment of pain interference in research and clinical practice in Germany.

## Supplementary Information

Below is the link to the electronic supplementary material.

Supplementary file1 (PDF 753 KB)

## Data Availability

The data that support the findings of this study are not openly available due to reasons of sensitivity and are available from the corresponding author upon reasonable request. Data are located in controlled access data storage at Charité - Universitätsmedizin Berlin.
