# Supplementary material for: Measuring PROMIS pain interference in German patients with chronic conditions: calibration, validation, and cross-cultural use of item parameters
Source: Qual Life Res. 2023 Jun 2;32(10):2839–52. doi: 10.1007/s11136-023-03446-6 (PMC10473994; doi:10.1007/s11136-023-03446-6)
Supplement: Supplementary file 1 — Supplementary file1 (PDF 753 KB) [file 11136_2023_3446_MOESM1_ESM.pdf]

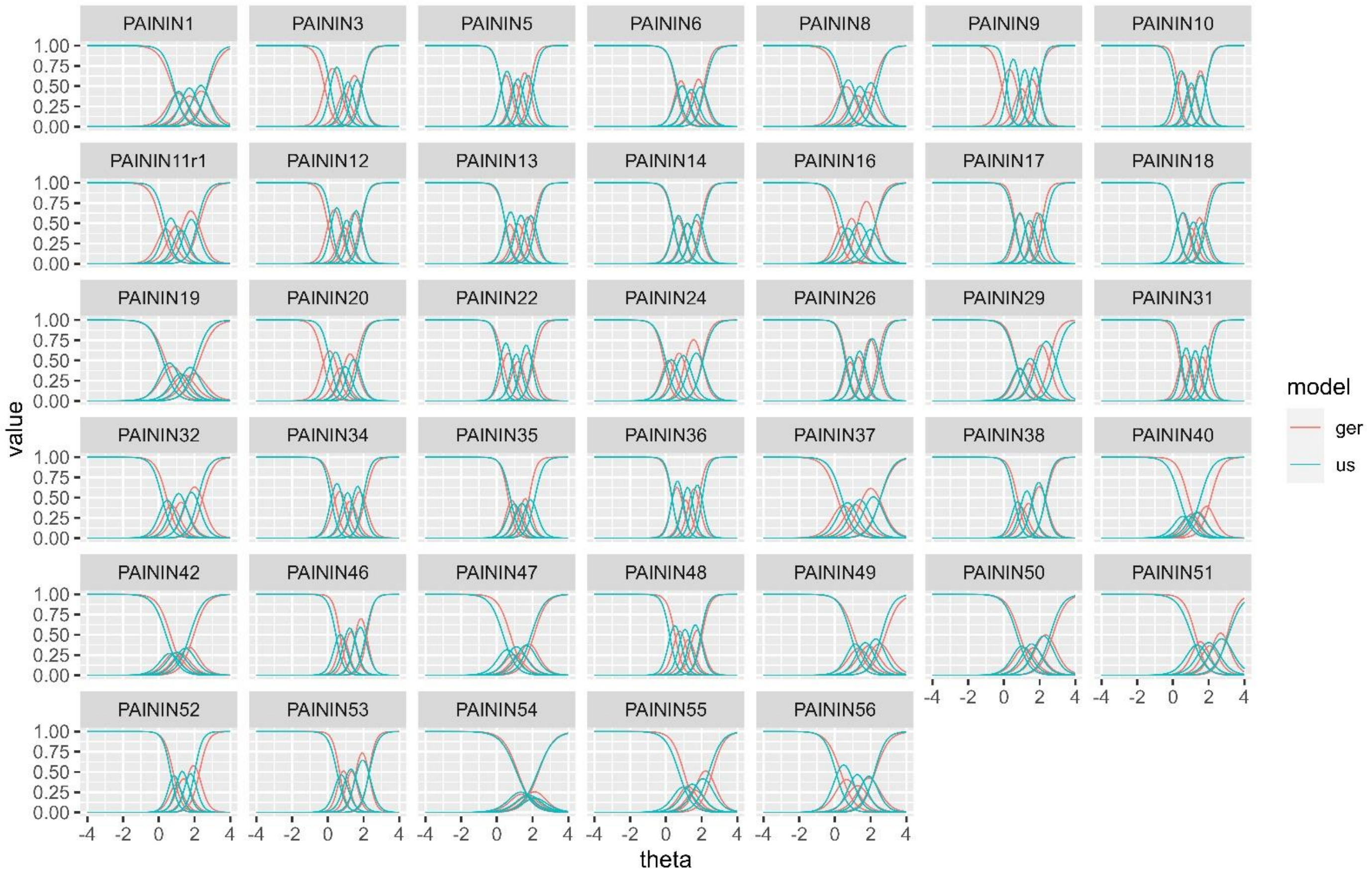

Figure S1 illustrates the item characteristic curves (ICCs) for each of the 40 PROMIS pain interference items. The green curves represent the original U.S. parameters, and the red curves represent the newly estimated parameters based on a German sample.

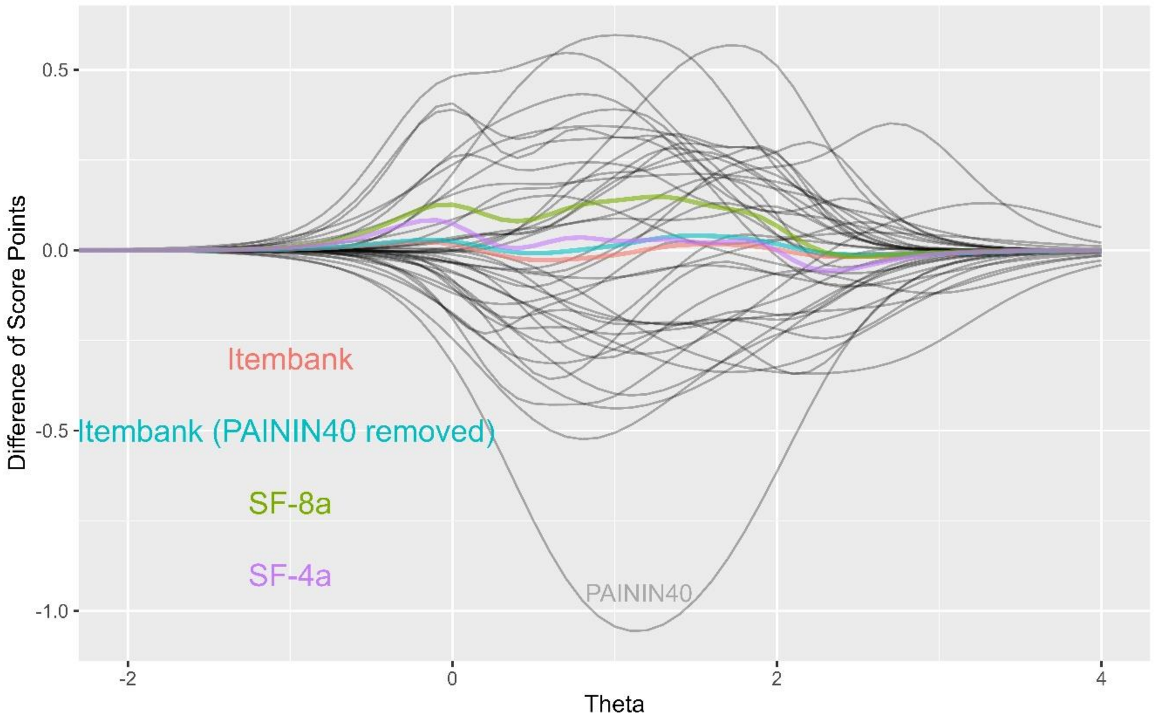

Figure S2 illustrates the difference of expected test scores between thetas based on the U.S. item parameters und thetas based on the German item parameters. Each black line represents a single item of the item bank. Colored lines represent combinations of items.
